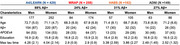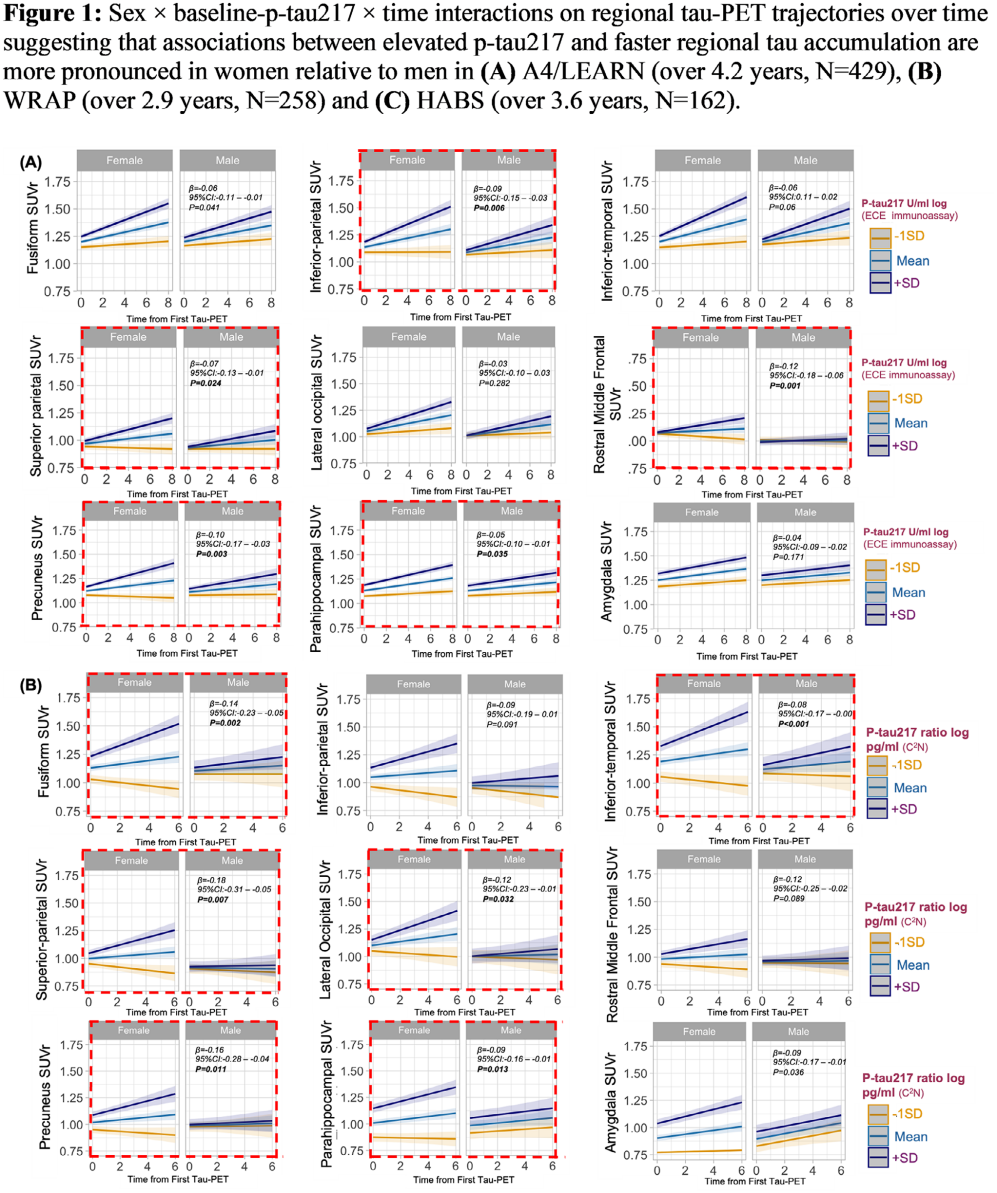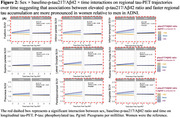# Sex Moderates Relationships Between *p*‐Tau217 and Longitudinal Tau‐PET: A Multi‐Cohort Study

**DOI:** 10.1002/alz70856_101667

**Published:** 2025-12-25

**Authors:** Gillian T Coughlan

**Affiliations:** ^1^ Massachusetts General Hospital, Harvard Medical School, Boston, MA, USA

## Abstract

**Background:**

Data suggest that women exhibit elevated insoluble tau aggregates relative to age‐matched men. Whether this sex difference is due to upstream soluble phosphorylated tau(*p*‐tau) is unclear. We examined whether sex and amyloid‐β(Aβ) predict baseline plasma *p*‐tau217 and whether baseline plasma *p*‐tau217 and *p*‐tau217/Aβ42 predict longitudinal tau positron emission tomography(PET) in a sex‐specific manner.

**Method:**

998 clinically normal individuals (mean‐age:71; 429 *APOE*ε4 carriers; 525 Aβ+; Table) from the Anti‐Amyloid Treatment in Asymptomatic Alzheimer's Disease trial, the companion Longitudinal Evaluation of Amyloid Risk and Neurodegeneration study(A4/LEARN), the Wisconsin Registry for Alzheimer's Prevention(WRAP), Harvard Aging Brain Study(HABS) and Alzheimer's Disease Neuroimaging Initiative(ADNI) had multiple tau‐PET scans and at least one measurement of ptau217. Average tau‐PET follow‐up time was 3.6 years (range=1.3‐8.9years. We used regression models of soluble *p*‐tau217 to examine cross‐sectional sex×Aβ interaction. Longitudinally, random‐effects models (participant‐specific intercepts and slopes) estimated the sex×baseline‐p‐tau217 time interaction on each of nine *apriori* tau‐PET regions (including rostral middle frontal gyri, fusiform gyrus, inferior temporal and parietal gyri, the superior parietal lobule, precuneus, lateral occipital cortex, parahippocampal and amygdala). All models adjusted age. ADNI used the *p*‐tau217/Aβ42 ratio.

**Result:**

In A4/LEARN, women exhibited elevated baseline *p*‐tau217 concentrations relative to men, particularly at higher neocortical‐Aβ(β=‐0.16, *p* = 0.008). No significant interactions were observed in the other cohorts. Longitudinal sex×baseline‐p‐tau217×time interactions were significant in five, six, four, and seven tau‐PET regions in A4/LEARN, WRAP, HABS and ADNI cohorts, respectively. Across all significant interactions, women showed worse tau trajectories than men at higher *p*‐tau217(A4/LEARN, WRAP, HABS; Figure 1) and *p*‐tau217/Aβ42(ADNI; Figure 2) concentrations. Covarying for *APOE*ε4‐status, baseline Aβ and baseline‐tau‐SUVR did not attenuate the longitudinal sex effects.

**Conclusion:**

These findings suggest that sex differences in tau proliferation may be exacerbated by upstream soluble *p*‐tau levels. Earlier therapeutic approaches reducing soluble *p*‐tau levels might be particularly important in women.